# IP-10 Levels as an Accurate Screening Tool to Detect Acute HIV Infection in Resource-Limited Settings

**DOI:** 10.1038/s41598-017-08218-0

**Published:** 2017-08-14

**Authors:** Lucía Pastor, Aina Casellas, Jorge Carrillo, Sergi Alonso, Erica Parker, Laura Fuente-Soro, Chenjerai Jairoce, Inacio Mandomando, Julià Blanco, Denise Naniche

**Affiliations:** 1 ISGlobal, Barcelona Centre for International Health Research (CRESIB), Hospital Clínic–Universitat de Barcelona, Barcelona, 08036 Spain; 2 AIDS Research Institute-IrsiCaixa, Hospital Germans Trias i Pujol, 08916 Badalona, Spain; 3 Institut Germans Trias i Pujol (IGTP), Hospital Germans Trias i Pujol, Universitat Autonoma de Barcelona, 08916 Badalona, Spain; 4 Centro de Investigação em Saúde da Manhiça (CISM), 1929 Maputo, Mozambique; 5 School of Paediatrics and Child Health, University of Western Australia, 6840 Perth, Australia; 6 Universitat de Vic - Universitat Central de Catalunya, 08500 Vic, Barcelona, Spain

## Abstract

Acute HIV infection (AHI) is the period prior to seroconversion characterized by high viral replication, hyper-transmission potential and commonly, non-specific febrile illness. AHI detection requires HIV-RNA viral load (VL) determination, which has very limited access in low-income countries due to restrictive costs and implementation constraints. We sought to identify a biomarker that could enable AHI diagnosis in scarce-resource settings, and to evaluate the feasibility of its implementation. HIV-seronegative adults presenting at the Manhiça District Hospital, Mozambique, with reported-fever were tested for VL. Plasma levels of 49 inflammatory biomarkers from AHI (n = 61) and non-HIV infected outpatients (n = 65) were determined by Luminex and ELISA. IP-10 demonstrated the best predictive power for AHI detection (AUC = 0.88 [95%CI 0.80–0.96]). A cut-off value of IP-10 ≥ 161.6 pg/mL provided a sensitivity of 95.5% (95%CI 85.5–99.5) and a specificity of 76.5% (95%CI 62.5–87.2). The implementation of an IP-10 screening test could avert from 21 to 84 new infections and save from US$176,609 to US$533,467 to the health system per 1,000 tested patients. We conclude that IP-10 is an accurate biomarker to screen febrile HIV-seronegative individuals for subsequent AHI diagnosis with VL. Such an algorithm is a cost-effective strategy to prevent disease progression and a substantial number of further HIV infections.

## Introduction

Acute HIV infection (AHI) is the period between the acquisition of human immunodeficiency virus (HIV) and the development of HIV-specific antibodies that define seroconversion^1^. AHI is characterised by high HIV viral replication and, in most cases, a transient non-specific febrile illness that typically occurs around 2 weeks after HIV acquisition^1, 2^. As a result of high viraemia in bodily fluids and high levels of genital shedding of the virus, individuals are considered hyper-infectious during AHI^3, 4^. In areas of high HIV incidence, this phenomenon could contribute greatly to fuelling the worldwide HIV pandemic^5^.

Despite the importance of early diagnosis and treatment to reduce onward transmissions^6, 7^ and prevent substantial irreversible immunological damage in gut associated lymphoid tissue^8, 9^, AHI represents a ‘window period’ during which persons infected with HIV are commonly undiagnosed^1, 10^. Routine second generation HIV-rapid test algorithms provide negative or indeterminate results for up to 6–8 weeks after infection^11^. During this time, HIV can only be diagnosed by detecting the presence of the virus itself^12^. The current gold-standard test for confirming viraemia is RT-PCR for plasma HIV-RNA^11^. However, technical and financial constraints make this technique very limited in low-income areas^1^ such as Sub-Saharan Africa (SSA), where the prevalence of AHI among febrile patients may reach 3%^13–15^.

As viraemia increases during AHI, there is a striking cascade response of inflammatory cytokines^2^. Significant efforts have been made to characterise host and viral proteins present during AHI aiming to identify biomarkers of progression or key pathological pathways that could be targeted to minimize HIV-induced immune damage over the course of infection^2, 16–19^. In the present study we sought to determine whether a single or a combination of biomarkers could differentiate individuals in AHI from non-HIV infected individuals within a population of patients reporting febrile symptoms at a rural hospital in Southern Mozambique. Once plasma level of IP-10 was identified as candidate biomarker, we assessed the cost-effectiveness of implementing it for AHI diagnosis in low-income settings.

## Results

### Characteristics of the study population

From the 2748 rapid-test seronegative patients who presented at Manhiça District Hospital (MDH) with reported fever during the screening period of the study, 61 (2.2%) were identified as AHI. Of the remaining HIV-RNA negative individuals, 65 (2.4%) were randomly selected as non-HIV-infected individuals with reported fever (NIF). As described in methods, of the 126 AHI and NIF controls, 95 individuals representing 75% of the study population were used for the model cohort. Median age of the model cohort was 23 years (IQR 20–30) and 68% were female. Eleven individuals (12%) had malaria at the moment of screening. Median HIV-RNA level in the AHI group was Log_10_ 6.8 copies/mL (IQR 6.1–7.4) and 70.4% patients showed a negative Western Blot for HIV-specific antibodies. There were no significant differences in age, gender or malaria status by study group (p > 0.1).

### Comparison of plasma inflammatory biomarker profile between AHI and non-HIV infected febrile controls

**Figure 1:**
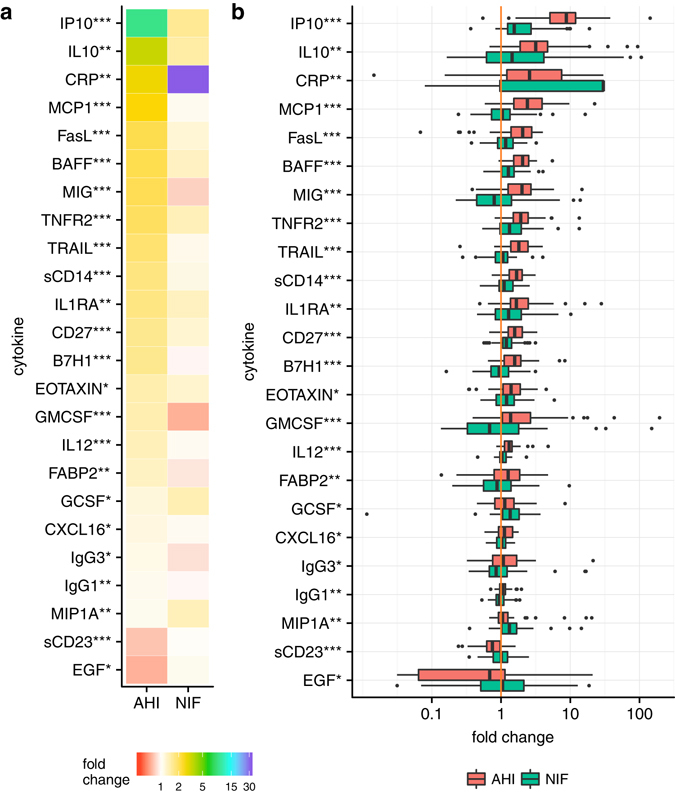
Normalized biomarker levels in AHI and NIF controls. (**a**) The levels of 24 plasma biomarkers are expressed as fold change compared to a reference level (defined in methods) for both AHI and NIF groups. Intensity of colour represents biomarker fold change and biomarkers are sorted by fold change value in the AHI group. Non-parametric significance of biomarker expression by study group is indicated as *** if P < 0.001, ** if P < 0.01, and * if P < 0.05. (**b**) Distribution of the normalized biomarker levels by study group. Results are expressed as fold change. Box as IQR, middle line as median, whiskers as Tukey values (1.5*IQR). The orange line corresponds to the null change compared to the reference level.

From a total of 49 quantifiable biomarkers, the median levels of 24 biomarkers were found to significantly differ between AHI and NIF individuals (p-value < 0.05). When comparing normalized biomarker levels between AHI and NIF individuals, the AHI group had a higher fold change for all the 24 biomarkers except CRP, GCSF, MIP-1alpha, sCD23 and EGF (Fig. 1a). IP-10 and CRP showed the most significant increase in the AHI and the NIF groups, respectively (p-value < 0.05). The distribution of the normalized biomarker expression by study group revealed overlap of interquartile range (IQR) values between AHI and NIF groups for most biomarkers; the exceptions being IP-10, MCP-1 and TRAIL (Fig. 1b). When assessing the correlation of the quantifiable biomarkers with the VL levels in the AHI individuals, IP-10 showed the strongest association (rho = 0.6, p < 0.0001).

### Designing a biomarker-based predictive model for AHI detection

Univariate analysis showed that 29 of the 49 biomarkers were associated with AHI (p-value < 0.2) and were included in the multivariate analysis. A total of 6 cytokines with a p-value < 0.05 were retained in the multivariate model (Table 1). IP-10, sCD14 and GMCSF were positively associated with AHI whereas CRP, MIP-1alpha and IL-10 were negatively associated; among these, IP-10 showed the strongest association with AHI (p-value = 0.0008).

**Table 1:** Adjusted multivariate logistic regression of biomarkers associated with AHI.

Variable		Coef.	(95% Conf. Interval)	p-value
Malaria status		3.21	(−0.81; 7.23)	0.1172
Sex	F	ref		0.7007
	M	0.40	(−1.66; 2.47)	
Age		−0.03	(−0.12; 0.06)	0.5253
LogIP10 (pg/mL)		5.68	(2.34; 9.01)	**0.0008**
LogCRP (ug/mL)		−1.99	(−3.68; −0.30)	**0.0209**
LogsCD14 (ug/mL)		14.20	(3.34; 25.07)	**0.0104**
LogGMCSF (pg/mL)		6.01	(1.44; 10.59)	**0.0100**
LogMIP1A (pg/mL)		−4.59	(−9.12; −0.06)	**0.0469**
LogIL10 (pg/mL)		−5.09	(−9.73; −0.45)	**0.0315**
*Intercept*		*−5.54*	(*−13.53; 2.45*)	*0.1743*

Coefficients per log_10_ cytokine unit increment, 95% confidence interval and p-value of the cytokines and confounders that entered into the model (p < 0.05) as described in methods.

**Figure 2:**
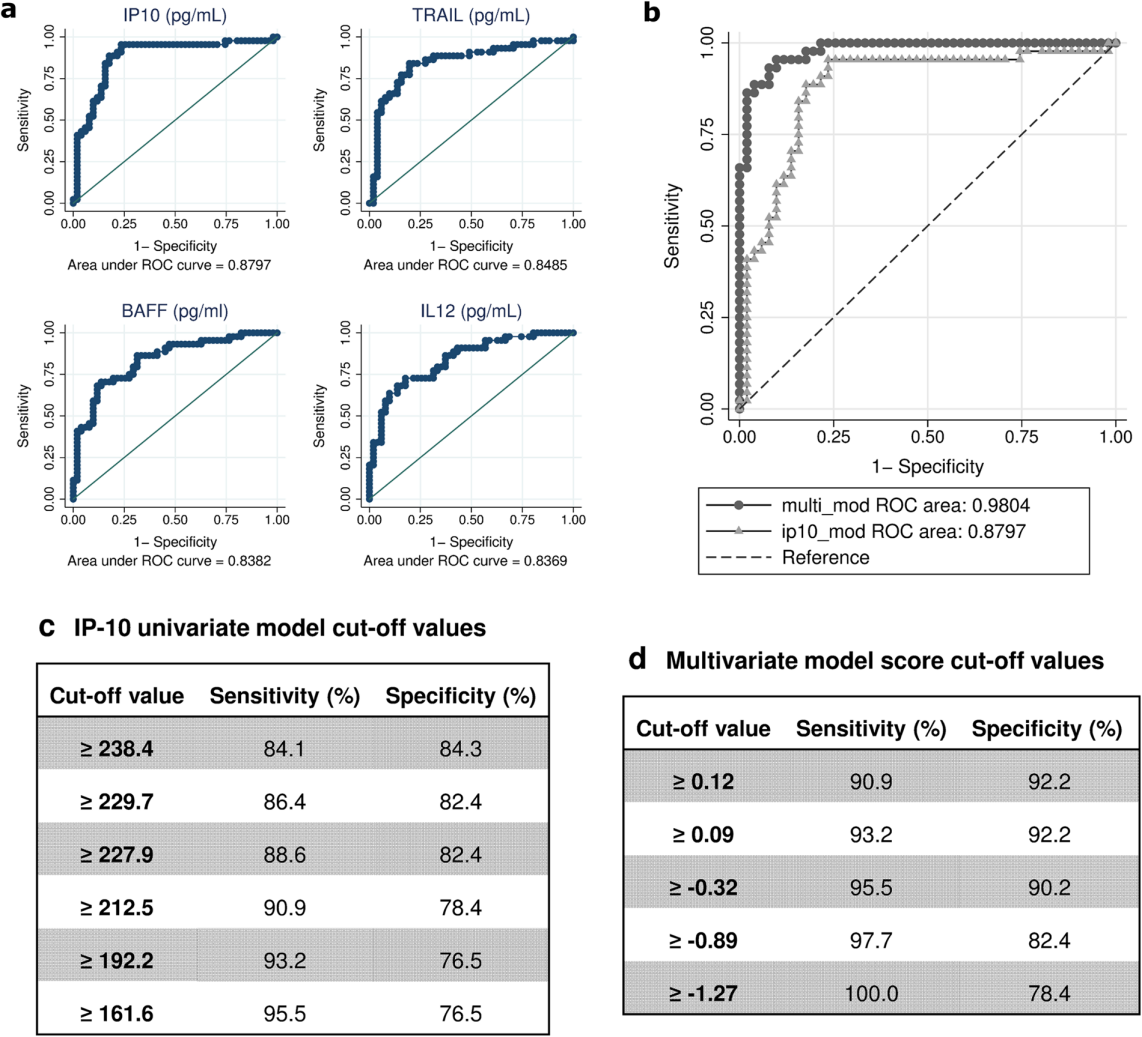
Performance of univariate and multivariate cytokine models in predicting acute HIV infection. (**a**) ROC curves for individual biomarkers with best AHI predictive accuracy (AUC > 0.8). (**b**) Comparison between ROC curves for univariate IP-10 model (AUC = 0.88) and adjusted multivariate biomarker model (AUC = 0.98). (**c**) IP-10 univariate model cut-off points (pg/mL) and (**d**) multivariate model score cut points with their respective sensitivity and specificity values.

Of the 29 biomarkers associated with AHI in univariate models, IP-10, TRAIL, BAFF and IL-12 cytokines showed the highest predictive power (Area under the curve (AUC) > 0.8) (Fig. 2a), where IP-10 demonstrated best accuracy as a single biomarker (AUC = 0.88 [95%CI 0.80–0.96]). Multivariate model adjusted by age, sex and malaria infection of the above-mentioned 6 cytokines further increased the classification performance (AUC = 0.98 [95%CI 0.96–1.00]) (Fig. 2b). Thus, IP-10 alone and the multivariate 6-cytokine model were identified as the best classification methods for AHI detection.

Receiver-operating characteristic (ROC) curves of the selected models were used to evaluate several cut-off values prioritizing the highest sensitivity (Fig. 2c and d). A cut-off of ≥ 161.6 pg/mL for the univariate IP-10 model provided a sensitivity of 95.5% (95%CI 85.5–99.5) and a specificity of 76.5% (95%CI 62.5–87.2) (Fig. 2c) for predicting AHI. A cut-off score of ≥−0.89 for the multivariate model provided a sensitivity of 97.7% (95%CI 88.0–99.9) and a specificity of 82% (95%CI 69.1–91.6) (Fig. 2d). Classification performance between the univariate and multivariate methods showed a substantial level of agreement for AHI detection with kappa-statistic of 0.66 (95%CI 0.51–0.81).

### Performance and validation of IP-10 as a single biomarker for AHI identification

**Figure 3:**
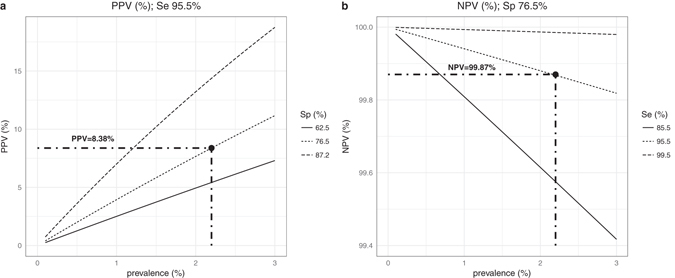
AHI Predictive power of the IP-10 model according to prevalence of AHI. (**a**) Positive predictive value (PPV) for varying AHI prevalence estimated for sensitivity = 95.5% and 3 different specificity scenarios according to the estimated confidence interval (Sp = 76.5% [95%CI 62.5–87.2]). (**b**) Negative predictive value (NPV) for varying AHI prevalence as estimated for specificity = 76.5% and 3 different sensitivity scenarios according to the estimated confidence interval (Se = 95.5% [95%CI 85.5–99.5]).

Given the greater simplicity of implementing a single cytokine assay, the univariate IP-10 model with a cut-off of ≥ 161.6 pg/mL was assessed for the AHI prevalence observed in the study cohort. We calculated positive and negative predictive values (PPV and NPV) using model sensitivity and specificity and their respective 95%CIs limits (Fig. 3). For an AHI prevalence of 2.2% in HIV-seronegative patients reporting fever, at a specificity of 76.5%, the PPV was 8.38% (Fig. 3a), and at a sensitivity of 95.5%, the NPV was 99.87% (Fig. 3b).

Using this IP-10 predictive model, 88% of the 17 AHI individuals and 86% of the 14 NIF controls in the validation sample set were correctly classified. These values fell within the 95%CI predicted for the sensitivity and specificity of IP-10, thus validating the model prediction power.

### Cost-effectiveness analysis of implementing an IP-10 rapid test to screen AHI cases in low-income settings

**Figure 4:**
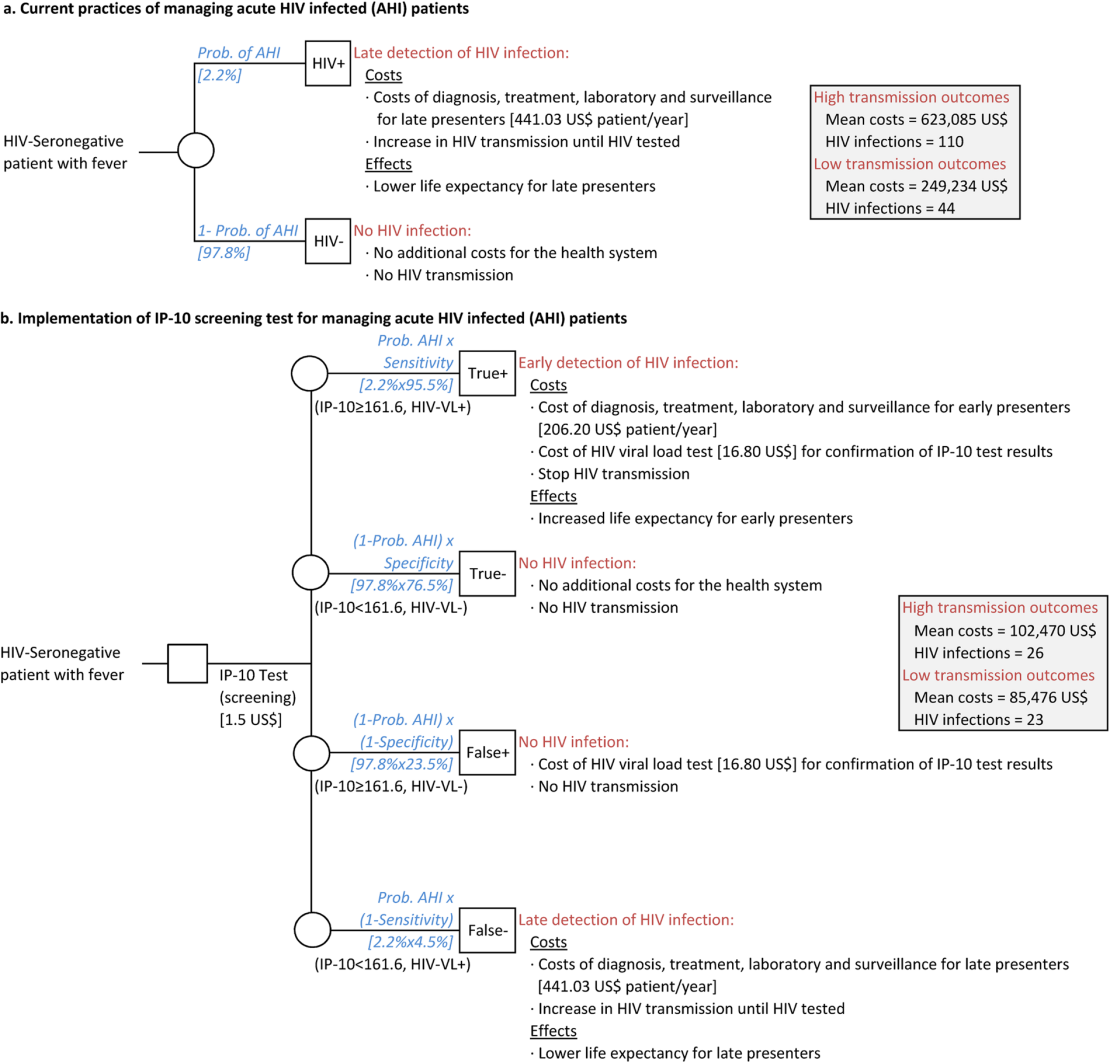
Graphic modelling of the cost-effectiveness analysis. Cost comparison between current practices which do not identify AHI (1a) and the implementation of a potential IP-10 rapid pre-screening test (1b) in seronegative febrile outpatients for AHI detection in a Sub-Saharan setting.

Projecting the observed AHI prevalence of 2.2% in a simulated cohort of 1,000 seronegative febrile individuals, 22 individuals would be expected to have AHI. Assuming a high and low transmission rate of 1:4 and 1:1, we would expect the 22 AHI to give rise to 88 or to 22 new infections, respectively. In a high transmission scenario, lifetime costs of the 110 HIV infections would total US$623,085 (Fig. 4a). In comparison, at a low transmission scenario, the mean cost for management of 44 HIV infections would be US$249,234.

The introduction of an IP-10 AHI pre-screening test would include additional costs for IP-10 tests and confirmatory VL determinations. With a sensitivity of 95.5%, IP-10 would identify 21 of the 22 AHI with the assumption that they would receive ART and thus not transmit the infection. However, 1 AHI would not be identified (false negative) and would generate 4 or 1 new infections depending on the transmission rate. For a high transmission scenario, the mean cost to the health system for a total of 26 HIV infected individuals would be US$89,618 (Fig. 4b) whereas for a low transmission scenario, the mean cost for 23 HIV infected individuals would be US$72,625. Thus, the cost savings are estimated to be US$6,351-8,410 per infection averted. Therefore, we estimate that the introduction of an IP-10 pre-screening test would save between US$176,609 and $533,467 and avert 21–84 new HIV infections per 1000 febrile seronegative outpatients.

## Discussion

We have demonstrated that among 49 inflammatory biomarkers assessed, the IP-10 cytokine has the highest accuracy in identifying individuals with AHI in a cohort of febrile HIV-seronegative individuals. A cut-off value for an IP-10 level of 161.6 pg/mL gave a sensitivity of 95.5% (95%CI 85.5–99.5) and a specificity of 76.5% (95%CI 62.5–87.2) in identifying AHI, which was confirmed in the validation sample set. The combination of IP-10 with other biomarkers showed a slightly higher prediction power for AHI detection but a substantial agreement was observed for classification performance between IP-10 and the multi-biomarker models. This suggests that IP-10 alone may provide a simple and precise way to screen febrile seronegative individuals for subsequent AHI diagnosis.

IP-10, also known as CXCL10, is a small cytokine belonging to the CXC chemokine family. IP-10 is produced as part of the innate immune response to viruses, bacteria, fungi and parasites^20^. At the initial stages of HIV infection, IP-10 has been shown to greatly increase prior to the development of clinical symptoms paralleling HIV-VL^19^. Previous data suggested that IP-10 levels were associated with immune activation^21^ and predictive of rapid disease progression^18, 22^, representing an earlier biomarker than CD4 T-cell counts or viraemia levels^18^. However, to our knowledge, the strong association between IP-10 and VL levels has not been assessed for its ability to identify AHI among seronegative individuals. Our results show that IP-10 can indeed be used to screen HIV-seronegative individuals with high predictive ability to differentiate AHI from other patients with undifferentiated fever.

IP-10 has been explored for its use as a prognostic or diagnostic marker for other infectious diseases such as malaria, where plasma IP-10 level is associated with fatal cerebral malaria^20^; hepatitis C, where plasma IP-10 has the power to predict liver damage^23^, or tuberculosis, where IP-10 levels differentiate between active and latent tuberculosis irrespective of HIV infection^24^. Interestingly, high IP-10 levels during HIV infection have been associated with increased susceptibility to malaria infection^25^, however this was not observed in our cohort, and our results have not shown an interference of malaria in AHI identification.

Different infections may be distinguished by distinct IP-10 thresholds, but additional research is necessary to explore the impact of malaria, tuberculosis and hepatitis C, common HIV comorbidities, on AHI-induced levels of IP-10. Parameters such as sensitivity, specificity and cut-off value also need to be optimized expanding to other populations affected by different HIV-subtypes or other prevalent co-infections, as well as to non-febrile seronegative individuals and pregnant women. Thus, although the IP-10 cut-off value of 161.6 pg/mL provided the highest sensitivity in our cohort, it may differ according to context. Importantly, if IP-10 accuracy for AHI detection is validated in non-febrile seronegative individuals, this biomarker could also be valuable for screening blood donors in low-income countries.

IP-10 is not a disease-specific biomarker^20^, and its potential use to identify AHI would require a second step of confirmation through point of care (POC) VL detection or other HIV-specific detection method. Here we suggest the use of IP-10, potentially developed as rapid test, as a pre-screening tool to discriminate AHI from other febrile seronegative outpatients in settings of high HIV incidence followed by a POC-VL. IP-10 showed a specificity of 76.5%, which would not allow IP-10 to be used as a standalone diagnostic test but represents a powerful screening tool to exclude non-HIV infected individuals. Furthermore, at estimated AHI prevalences below 5%, the NPV for the IP-10 remains above 99%, thus ensuring that a low number of AHI cases would go undiagnosed. In low-income countries, where the cost of individual as well as pooled HIV-VL determinations or similar antigen-based assays is prohibitive and the logistics for VL implementation remain highly complex, this powerful tool would make AHI screening affordable and feasible, allowing early ART in these individuals and preventing a substantial numbers of new HIV infections.

Current practices in Mozambique and similar SSA settings use HIV rapid test serology, which detects infection from 6 to 8 weeks post-transmission onward, and AHI screening is not conducted. The implementation of an IP-10 test to pre-screen febrile seronegative patients could save from 176,609 to 533,467 US$ in treatment and diagnostic costs to the health system per 1,000 patients tested, while averting up to 84 new HIV infections. Hence, despite the necessity of confirming positive diagnosis with a POC-VL or similar, the introduction of a potential IP-10 rapid test as a pre-screening tool would be an important cost-saving strategy compared to leaving AHI undiagnosed. We used a public negotiated cost of $16.80 as a POC-VL instead of employing a pooling or individual VL strategy because same day return of results has been shown to accelerate ART initiation^26^. However, for current laboratory-based HIV-VL there are major pricing discrepancies across countries^27, 28^. Recently, MSF reported that comprehensive costs including human resources, sample collection, reagents and consumables ranged from US$24.90 to 43.42 in the five Sub-Saharan Africa countries surveyed in 2013 (Kenya, Lesotho, Malawi, Swaziland, and Zimbabwe)^29^. We assumed a best case scenario but using these values would increase the cost savings. We assumed a cost of an IP-10 rapid test of $1.50 based on commercially available costs for CRP rapid test employed in the management of undifferentiated fever^30^. Both CRP and IP-10 are inflammatory proteins with similar costs for detection by ELISA assays purchased at market price. However, the cost-effectiveness model was neither probabilistic nor dynamic. Importantly, we disregarded the cost of HIV-associated comorbidities in the simulated cohort which, if included, would most likely increase the cost-effectiveness of the IP-10 followed by POC-VL intervention.

Finally, as it has been amply demonstrated, early ART stops progression to AIDS^31, 32^, diminishes the viral reservoir^33^ and early immunological damage^9^, as well as reducing further transmissions^7^. Since the World Health Organization updated the HIV recommendations in 2015^34^, many SSA countries are rolling out “test and start” programs to initiate ART regardless of CD4 counts. This implies expansion of serological HIV testing strategies but also places AHI detection in the spotlight. Although the development of POC-VL systems has rapidly advanced, implementation costs remain elevated. Confronted with the sheer volume of patients requiring VL determinations for ART monitoring in many SSA countries, health services will not be able to implement individual or pooled VL for AHI diagnosis among febrile seronegative outpatients in the near future^35^. A new approach for AHI detection using an affordable IP-10 pre-screening tool to reduce the number of VL determinations necessary by 75%, may be an opportunity to bring AHI diagnosis to low-resource high HIV-burden settings.

In conclusion, we demonstrated that a screening based on IP-10 levels is an accurate and cost-effective strategy to detect AHI in HIV-seronegative patients with undifferentiated fever. This algorithm renders AHI diagnosis feasible in low-income settings and by treating these individuals a substantial number of progressions to AIDS and further HIV transmissions could be averted.

## Methods

### Study population

The study population was enrolled between 2013 and 2014 at the MDH in Mozambique. The present analysis is a sub-study of a prospective cohort of primary HIV-infected adults enrolled^15^ and followed up for 12 months in the Gastro-intestinal biomarkers in AHI Mozambican Adults study (GAMA). This study was approved by local institutional review boards at Barcelona Clinic Hospital (2011/6264) and by the Ministry of Health of Mozambique (461/CNBS/12). All methods were carried out in accordance with the relevant guidelines and regulations. Written informed consent was obtained from patients prior to participation.

### HIV diagnosis and biomarker quantification

Technical information and procedures regarding HIV diagnosis, clinical follow up, HIV-specific antibody determination and biomarker quantification have been previously described^15^. Individuals presenting to the outpatient clinic of MDH with reported fever, flu-like symptoms or malaria suspicion were included in the AHI group of this sub-study if they were HIV-rapid test seronegative and HIV-RNA positive. A control population was established by random selection among NIF and their screening samples were employed to compare cytokine expression levels with the AHI group. At an additional visit, non-febrile HIV-uninfected controls (n = 58) provided samples that were used to establish biomarker reference levels in the absence of fever illness^15^. HIV-specific serology was performed by Western Blot. Plasma levels of non-specific antibody isotypes were quantified by an in house ELISA. Multiplex cytokine profiling was performed in plasma samples with particular interest in inflammatory cytokines, chemokines, hematopoietic factors and biomarkers of intestinal damage. Determinations were performed by ELISA commercial assays or Luminex^15^.

### Statistical analysis

Of the 126 individuals included in this cohort, 75% were used for the data analysis and training set (n = 95) and 25% for model validation (n = 31). Proportions were compared using chi-square test. Biomarker values were log transformed for a better adjustment of skewed data. In order to normalize biomarker levels, fold change was calculated as the ratio of the biomarker level in individuals reporting fever over the median value of the non-febrile HIV-uninfected reference group. Distributions of biomarkers in AHI and NIF groups were compared by Mann Whitney U-test. Spearman’s correlation was used to assess the strength of relationship between continuous variables.

In order to assess the predictive ability of differentially expressed biomarkers to discriminate between AHI and NIF individuals, logistic regression with penalized likelihood was performed^36^. A multivariate model was constructed using backward step-wise elimination with an inclusion criterion of p-value < 0.2 in univariate analysis. Age, gender and malaria infection were retained as potential confounders in the multivariate model because of their previous association with alterations in the cytokine expression levels^37–39^. ROC curves from univariate and adjusted-multivariate models were compared for the best prediction^40^. The level of agreement between the classification methods was assessed by the κ-statistic^41^. Data were analysed using R-3.2.4 software (R Core Team 2016) and Stata Statistical Software: Release 14 (StataCorp 2015. College Station, TX).

### Cost-effectiveness analysis

A deterministic decision analysis was used to estimate the cost-effectiveness of introducing a potential IP-10 rapid test to screen febrile HIV-seronegative patients attending a health facility compared to current practices in Mozambique, which at present include only HIV antibody-based rapid testing, and thus no AHI identification. Health effects and costs to the health care provider associated with HIV/AIDS were estimated for a simulated cohort of 1,000 febrile seronegative patients 20 years of age. Economic model outcomes were determined using the AHI prevalence observed in this study and the sensitivity and specificity of IP-10 obtained in the univariate model. Health effects were expressed as the number of averted infections. Costs per person-year to the health system included: (1) first-line ART drugs and their delivery (assuming complete linkage-to-care); (2) laboratory tests and patient surveillance; and (3) treatment of opportunistic infections including tuberculosis prophylaxis^42^. A unit cost of US$1.50 for a hypothetical IP-10 rapid test was based on costs for the CRP rapid test, similarly employed to assist diagnosis among patients with undifferentiated fever^30^. A cartridge-based nucleic acid amplification test (GeneXpert; Cepheid, Sunnyvale, CA, USA) was included as a POC-VL confirmation assay with a unit cost of US$16.80, according to FIND negotiated prices for eligible countries^43^. Costs per HIV-infected individual were different between early (CD4 T-cells count > 200) and late presenters (CD4 T-cells count =< 200) and both were multiplied by their estimated life expectancy to model life-long treatment costs^44^. Costs were expressed in US$, inflation-adjusted (3%) and discounted (3%). Costs and effects were estimated for two different HIV transmission scenarios^45^. This economic analysis represents a static model where transmission only occurs from our cohort of 1,000 individuals to a number (n) of other individuals, where n can assume the values of 1 or 4 (low and high transmission scenarios, respectively).

### Data Availability

The datasets generated during and/or analysed during the current study are available from the corresponding author on reasonable request.
